# Mismatch Negativity Responses to Different Auditory Attributes in Normally Developing Infants and Children

**DOI:** 10.7759/cureus.33163

**Published:** 2022-12-31

**Authors:** Sangeeta Gupta, Anchala Bhardwaj

**Affiliations:** 1 Physiology, All India Institute of Medical Sciences, Gorakhpur, IND; 2 Pediatrics, All India Institute of Medical Sciences, Gorakhpur, IND

**Keywords:** amplitude, latency, deviant, standard, auditory, paradigms, mismatch negativity

## Abstract

Introduction

Mismatch negativity (MMN) is a change-specific component of the event-related potentials that is elicited by an irregularity in repetitive auditory stimulation. As it is developmentally stable and can be measured in the absence of the participant’s attention, it can be a valuable method for assessing auditory discrimination in infants and young children. The classic MMN paradigm involves tone frequency as the mismatching attribute. Multi-feature MMN paradigms which involve different auditory attributes can assess discrimination abilities in a wider group of disorders. The study aimed to report standardised MMN values obtained with MMN paradigms including several auditory attributes to extend the clinical applicability of the test in infants and young children.

Methods

MMN responses were recorded in 42 normal infants and young children (2 months to 5 years) with multi-feature MMN paradigms. MMN variables in different trials were compared by one-way ANOVA. Pearson’s correlation coefficient and independent sample t-test were performed for finding an association with the age and gender of the participants respectively. P<0.05 was considered as statistically significant.

Results

MMN amplitude exhibited statistically significant differences in different MMN paradigms (p<0.05). An increase in the degree of standard and deviant differences and double deviant responses also resulted in larger MMN. MMN latency variation in the trials was not statistically significant. The age and gender of the participants did not influence the MMN variables with statistical significance.

Conclusion

MMN paradigms with different auditory attributes report significant amplitude variations. Multi-feature MMN paradigms can optimize the clinical applicability of the test and can determine the profile of different auditory discrimination abilities.

## Introduction

Mismatch negativity (MMN) is generated as a frontal/fronto-central negative deflection in the human event-related potential. It is a pre-attentive response elicited by changes in repetitive auditory stimulation. MMN peaks within 100-200 ms post-stimulus, and is usually identified as a difference between the event-related potential elicited by a high-probability standard and that elicited by a low-probability deviant stimulus [1].

MMN studies involve the presentation of frequent standard sound and an infrequent deviant sound in passive oddball paradigms. The mismatching stimuli can differ on any discriminable auditory dimension such as pitch, duration, intensity, or location. MMN is obtained by subtraction between the standard and deviant responses [2-4]. The most common attribute studied for detecting discrimination is the pitch of the stimuli [5-8].

MMN can be used to evaluate discrimination acuity as the peak latency and the peak amplitude varies with the magnitude of the difference between standard and deviant stimuli. It represents an objective index for auditory discrimination. Objective audiometry measures auditory detection while accuracy in detecting the sound-discrimination abilities is provided by MMN responses [9].

MMN can be elicited without special task requirements, independently of the subject’s motivation and in the absence of attention, during sleep. This feature makes it particularly suitable for testing infants and newborns [10]. Also, unlike many other components of event-related potentials (ERPs), the MMN is developmentally quite stable and can be obtained even from pre-term infants [11]. This also makes it possible to assess central auditory processing abilities in the younger age groups.

MMN is characterized by a myriad of traits that distinguishes it from other exogenous and endogenous event-ERP components which are sensitive to irregularities in repetitive stimulation, such as the P1, N1, P3a, P3b, or N400. This special combination of features makes MMN an attractive tool for studying various aspects of central auditory information processing [12].

In children with dyslexia, the amplitude of MMN elicited by a change in tone frequency is reduced considerably [13]. It has been utilized to evaluate the improvement of auditory discrimination after the installation of a cochlear implant. MMN study of cortical auditory discrimination in an adult and child cochlear implant population found a correlation between auditory performance and the presence of this negative wave [14,15]. It has been found to be valuable in assessing language and auditory processing disorders with reading and writing disorders and dyslexia, with stuttering and with aphasia [16-22].

Notwithstanding its valuable and wide clinical applicabilities in infants and children, the variability in its measurements and the procedure protocols necessitate the attainment of standardized values of MMN latencies and amplitudes. Moreover, most studies evaluate the MMN responses to pitch discrimination [23-25]. Some autistic spectrum disorders, however, have been associated with differences between intensity deviants in addition to frequency deviants [26,27]. A particular auditory feature is affected in different clinical populations. Hence, obtaining MMN responses to more than one auditory attribute would enable a better evaluation of discrimination abilities and their abnormalities. Very few studies, however, have recorded and demonstrated MMN responses to intensity deviation [28].

The present study attempts to assess the MMN responses in infants (typically developing) and young healthy children in response to deviations in different auditory features (duration, intensity and frequency) during the stimulus presentation. It allows the evaluation of specific MMN alterations in a variety of clinical conditions that is feasible for clinical work to a great extent. Standardised MMN latencies and amplitudes in the studied age group can thenceforth be optimally applied in various phonological disorders and developmental disabilities in infants and children resulting in wider application in the specific group of children. Hence, the purpose of the present study was to delineate the values of the latencies and amplitudes of MMN in infants and young children with normal auditory thresholds and without auditory processing disorders and to outline the values with respect to age, gender and different auditory attributes studied.

## Materials and methods

Forty-two healthy infants and young children (22 females and 20 males) in the age group between 2 months and 5 years (60 months) were recruited by homogenous purposive sampling method. The study was conducted in the Neurophysiology Laboratory, Department of Physiology, All India Institute of Medical Sciences (AIIMS) Gorakhpur. Approval for conducting the study was attained from the institutional ethics committee (Institutional Human Ethics Committee (IHEC), All India Institute of Medical Sciences, Gorakhpur, India, Reference number: IHEC/AIIMS-GKP/BMR/60/2022).

It was an analytical cross-sectional study. All the participants presented normal auditory thresholds (with respect to infants and children). Auditory brainstem evoked response (ABER) threshold ≤40 dB nHL (decibels normalized hearing level) bilaterally in infants while ≤30 dB nHL in children (>1 year) [29,30].

Infants with normal developmental milestones (physical, language, cognitive and social/emotional milestones) were included in the study. For children, learning difﬁculties, language, speech, hearing, otological history, and family history of hearing problems of the participants were collected. Children with cognitive disorders, speech delay, learning disorders were excluded. Infants and children with genetic or craniofacial abnormalities were also excluded.

Informed written consent from the parents as well as filled and signed minor assent forms were obtained before conducting the tests. Participants were given appropriate instructions regarding the procedure that would be performed. Auditory assessment of the infants and young children was performed by ABER tests [31].

Recording of mismatch negativity responses

MMN was recorded on Neuro-MEPω electromyography and EP Digital Neurophysiological System software (M/S Neurosoft Ltd, Ivanovo, Russia) in Neurophysiology Laboratory, AIIMS, Gorakhpur by a single channel recording performed in a quiet environment.

Before recording the responses, the subject was prepared for the test. The skin was cleaned with a Nuprep-skin preparation gel and with gauze. Subsequently, gold-plated cup electrodes were placed along with the application of Ten20 electrode paste and micropore surgical tape. Electrode impedance was kept below 5 Ω in each lead. An International 10-20 system was used to apply the electrodes [32]. The Active (non-inverting) electrode at Vertex (Cz), reference (inverting) at the ipsilateral mastoid while the ground/common electrode was placed at low forehead (Fpz) [33].

MMN was recorded using an oddball paradigm in which a series of identical or “standard” stimuli and different or “deviant” stimuli were presented. The probability of the deviant sound was 0.20. MMN was recorded and marked as a difference waveform obtained by subtracting the average ERP from the standard stimulus from the average ERP to the deviant stimulus [2].

A visual distraction task in the form of watching a movie (with low or no sound levels)/reading a book was used while recording MMN in older children. The equipment had a high pass ﬁlter of 0.5 Hz, low pass ﬁlter of 35 Hz, and notch of 50 Hz kept on and the time window was 700 ms [2]. The auditory stimuli (tone-burst stimuli with condensation polarity) were presented in monaural mode, ﬁrst in the left ear and then in the right ear through headphones (TA-01) placed on both ears.

The deviant tones differed from the standard tones in multiple auditory attributes (duration, frequency and intensity) in separate trials:

Recording of single deviant MMN responses-frequency variation (one frequent standard and one rare deviant tone with variation in frequency):

MMN for frequency deviation was recorded by presenting first a series of standard stimuli (frequency: 1000 Hz) and deviant stimuli (frequency: 1100 Hz) with 80 dB SPL (decibels sound pressure level) intensity (Single deviant response-frequency). Stimulus duration had a minimal difference (90 ms for standard while 89 ms for deviant).

Recording of double deviant MMN responses (one frequent standard and one rare deviant tone with variation in frequency and duration):

A series of standard stimuli (frequency: 1000 Hz and duration: 90 ms) and deviant stimuli (frequency: 1500 Hz and duration: 65 ms) with the same intensity (80 dB SPL). Hence, deviant varied in two auditory attributes (Double deviant response-I). Subsequently, to record the responses the following increment in the magnitude of the auditory attribute of the deviant stimuli, a series of standard stimuli (frequency: 1000 Hz and duration: 90 ms) and deviant stimuli (frequency: 2000 Hz and duration: 49 ms) with the same intensity (80 dB SPL) was presented (Double deviant response-II).

**Figure 1:**
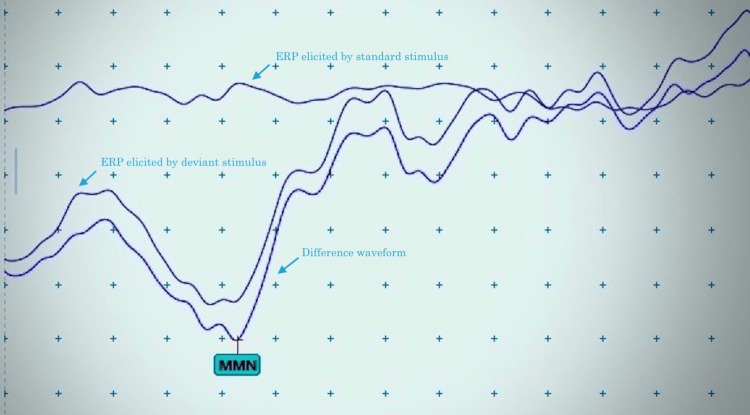
Mismatch negativity waveforms at CZ (reference: mastoid) recorded in a one-year-old normal female. Sweep: 50 ms/division, Sensitivity: 10 µv/division, standard stimuli with intensity 80 dB SPL while deviant stimuli with 90 dB SPL, probability of the deviant sound: 0.20, Interstimulus interval: 700 ms) ms: milliseconds; µv: microvolts; dB SPL: decibels sound pressure level; ERP: event-related potential

Recording of Single deviant MMN responses-intensity variation (one frequent standard and one rare deviant tone with variation in intensity):

MMN for intensity deviation was recorded by presenting a series of standard stimuli with intensity of 80 dB SPL and deviant stimuli with 90 dB SPL intensity (single deviant response for intensity-I). The frequency of both standard and deviant stimuli was 1000 Hz. Thereafter, deviant stimuli varied as 100 dB SPL followed by 110 dB SPL in the subsequent trials (about a 10% increment in deviant stimuli frequency) (single deviant response for intensity II and III, respectively). Difference waveform for each set of stimulation was generated (automatically) (Figure 1). Two hundred responses were averaged and two records were superimposed to ensure the reproducibility of the traces. MMN latencies (in milliseconds) from auditory stimulus onset to maximum peak; and amplitudes (in microvolts) from baseline to maximum peak were measured from different waveforms for each trial.

Statistical analysis

The data were expressed as mean ± standard error of the mean (SEM). One-way ANOVA was applied to compare the mean values of MMN latencies and amplitude between MMN responses obtained in various trials (for single deviant responses for frequency and intensity attributes as well as for double deviant responses). An Independent sample t-test was performed for comparing the values in relation to gender. Pearson’s correlation coefficient test was applied for studying the correlation between the age of the participants and the MMN latency and amplitude values. A level of 5% (p < 0.05) was considered as the criterion for statistically significant differences. The analyses were performed in the SPSS software version 28.0.0 (IBM Corp., USA).

## Results

**Figure 2:**
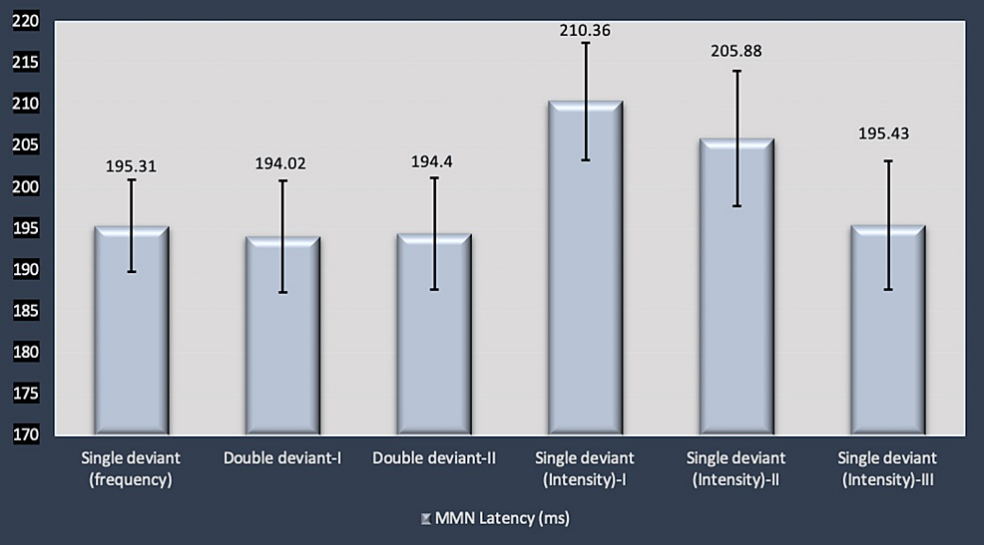
Comparison of MMN latencies (ms) (mean ± SEM) in MMN responses obtained with different trials. Single deviant (frequency): standard stimuli (frequency: 1000 Hz) and deviant stimuli (frequency: 1100 Hz) with 80 dB SPL; double deviant-I: Standard stimuli (frequency: 1000 Hz and duration: 90 ms) and deviant stimuli (frequency: 1500 Hz and duration: 65 ms) with same intensity (80 dB SPL); double deviant-II: standard stimuli (frequency: 1000 Hz and duration: 90 ms) and deviant stimuli (frequency: 2000 Hz and duration: 49 ms) with same intensity (80 dB SPL); single deviant for intensity I, II and II: standard stimuli with intensity 80 dB SPL while deviant stimuli with 90 dB SPL, 100 dB SPL, 110 dB SPL, respectively). ms: milliseconds; SEM: standard error of mean; MMN: mismatch negativity

MMN was recorded in 42 healthy infants and children (mean age: 24 ± 2.53 months) with single deviant and double deviant paradigms with different auditory dimensions. MMN variables were compared among various trials by one-way ANOVA. Mean latency values showed a decreasing trend in the trials with single deviant (intensity variation) when an increment in the magnitude of difference in the intensity was presented (Figure 2). The decrease in latency, however, was not statistically significant (p=0.37). Overall comparison of the mean latency values in all trials including single deviant paradigms for frequency and intensity deviants as well as double deviant responses were not found to be statistically significant (Figure 2).

**Figure 3:**
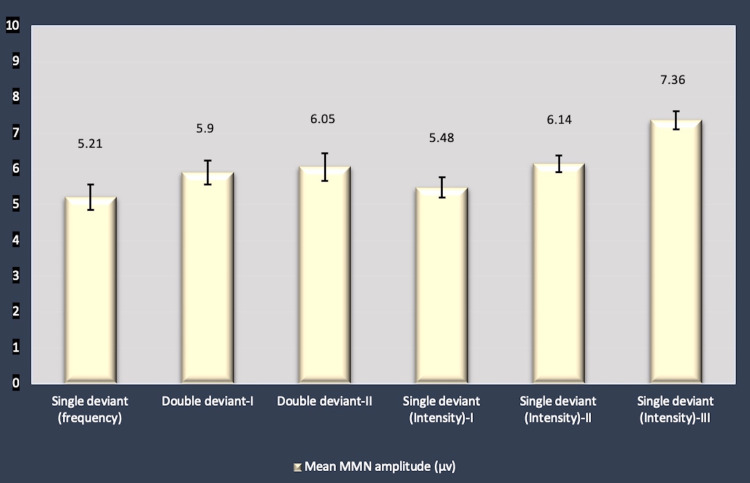
Comparison of MMN amplitudes (µv)((mean ± SEM) in MMN responses obtained with different trials. Single deviant (frequency): standard stimuli (frequency: 1000 Hz) and deviant stimuli (frequency: 1100 Hz) with 80 dB SPL; double deviant-I: standard stimuli (frequency: 1000 Hz and duration: 90 ms) and deviant stimuli (frequency: 1500 Hz and duration: 65 ms) with same intensity (80 dB SPL); double deviant-II: standard stimuli (frequency: 1000 Hz and duration: 90 ms) and deviant stimuli (frequency: 2000 Hz and duration: 49 ms) with same intensity (80 dB SPL); single deviant for intensity I, II and II: standard stimuli with intensity 80 dB SPL while deviant stimuli with 90 dB SPL, 100 dB SPL, 110 dB SPL, respectively. µv: microvolt; SEM: standard error of mean; MMN: mismatch negativity

MMN amplitude comparison among the trials revealed greater amplitude values for MMN obtained with intensity deviant responses. Overall comparison of mean MMN amplitude for values obtained from all trials demonstrated statistically significant differences (p<0.01) (Figure 3) with a statistically significant increase in the MMN amplitude in single deviant response for intensity-III as compared to other sets of the experiment (posthoc tests for comparison within the groups). Also, an increasing trend of amplitude with san increase in the magnitude of stimulus variation was found in the intensity deviant responses (p<0.01) (one-way ANOVA) (Figure 3). A similar increase in the MMN amplitude with an increase in the magnitude of stimulus variation was also noted for double deviant responses but with no statistical significance (Figure 3).

Gender comparison did not influence the latency and amplitudes with statistically significant differences (Table 1). The negative correlation obtained with age while comparing the latencies did not reach the statistical significance level (r=-0.12, p=0.45). Amplitude values increased with age but with no statistical significance (r=0.22, p=0.16) (Table 2).

**Table 1:** Comparison of mean MMN latencies and amplitudes between the genders (MMN responses for single deviant (frequency) paradigm). *Independent sample t-test ms: milliseconds; µv: microvolt; SEM: standard error of mean; MMN: mismatch negativity

MMN variables	Males (n=20)	Females (n=22)	p-value*
Latency (Mean ± SEM ms)	195.50 ± 6.19	195.14 ± 9.14	0.97
Amplitude (Mean ± SEM µv)	4.65 ± 0.44	5.73 ± 0.53	0.13

**Table 2:** Mean MMN latencies and amplitudes in relation to age (for single deviant (frequency) paradigm) MMN: mismatch negativity

MMN variables	Correlation coefficient value (r)	p-value
Latency	-0.12	0.45
Amplitude	0.22	0.16

Comparison between gender and correlation with age was computed for the single deviant paradigm (frequency deviant) [2].

## Discussion

MMN component in human ERP is elicited by any discriminable change in auditory stimulation. The main intracerebral sources are suggested to be located in the auditory cortices of the temporal lobes. Contribution from the right frontal cortex has also been suggested in its generation [34]. It is thought to represent an automatic cerebral process which can detect change even in the absence of a participant’s attention. This feature enables the assessment of auditory discrimination among infants and young children. Variation of MMN peak latency and the peak amplitude with the magnitude of the difference between standard and deviant stimuli makes it a useful tool to evaluate auditory acuity for discrimination.

The classic paradigm for recording MMN involves the presentation of a series of identical auditory stimuli (standard) with occasional mismatching (deviant) stimuli which is mostly frequency. MMN can, however, be recorded with the deviant stimuli differing on other discriminable auditory dimensions such as duration, intensity or location. Multi-feature MMN paradigm can assess discrimination abilities in a wider group of disorders. Among children with an autistic spectrum of disorders larger MMNs for intensity and smaller MMNs for frequency changes than typically developing children were obtained. Other deviant stimuli could not find any MMN group differences in the studies [26,27].

MMN responses in different sets with deviant stimuli differing on the discriminable auditory feature as single or double deviant responses were obtained in the present study. MMN was elicited in all the participants. Tone-burst stimuli employed in the present study have been suggested to be more reliable as compared to the speech stimulus in children when intraindividual variability is concerned [35].

The mean MMN latency obtained with the classic paradigm (frequency variation) in the present study was 195.31±5.55 ms. The latency values comply with those in previous similar studies with infants and young children [6,8,23,36]. MMN latency has been reported to be slightly longer in infants [11]. The studies conducted on older children, however, report decreased latency values (<190 ms) [23,37].

Double deviant responses demonstrated similar values as compared to the classic paradigm with frequency variation (194.02±6.72). The above finding is supported by the previous study by Schröger (1998) which stated that the latency of MMNs to two-dimensional deviants was not different from that of the MMNs to one-dimensional deviants [3].

The intensity deviant responses (Single deviant response with intensity deviant-I), however, demonstrated greater latency values (201.35 ±7.08 ms) as compared to other sets of the experiment, but without statistical significance. Not many studies in the past have evaluated MMN responses in infants and children with intensity deviants [26-28].

MMN latency is considered to reflect the nature and difficulty of the standard-deviant comparison process. It is influenced by the discrimination difficulty. MMN latency is reduced where the two tones are more clearly distinct and will extend to as long as 200-300 ms in the case of barely discriminable differences [9,38]. Similar findings were observed in the present study for MMN latency values in single deviant responses for intensity (I, II and II). MMN latency decreased with an increase in the stimulus variation. The difference, however, was not statistically significant (p=0.37) (Figure 3).

The mean MMN amplitude was 5.21 ± 0.35 µv in the classic MMN paradigm. It has been reported that when a deviant is different from the standard in more than one stimulus dimension, MMN is larger than when it differs in one feature only [3].

Larger MMN in double deviant responses (6.05 ± 0.39 µv) as compared to single deviant response (frequency) in the present study were in line with the above findings (p<0.01) (double deviant response-II). The maximum values of MMN amplitudes, however, were recorded for the intensity deviant response-III (Figure 3). An increasing trend (increase in the MMN amplitude with an increase in the magnitude of the stimulus (10% successively)) was evident with statistical significance, in intensity deviant responses (p<0.01) (Figure 3).

MMN amplitude is also considered to reflect some quantification of discrimination difficulty [4]. MMN is larger when the difference between the standard and deviant is more marked, whether this is due to a greater degree of physical difference between the tones or concurrent deviation on multiple stimulus dimensions [39]. The present study also demonstrated an increase in amplitude when the degree of standard and deviant differences increased. Also, multidimensional variation in deviant stimuli (double deviant responses) reported greater amplitude in our study.

The age of the participants demonstrated a decrease in the latency when correlated with mean MMN latency from the classic MMN paradigm and increase in the amplitude with age. No statistical significance obtained in the present study conforms to the findings in previous similar studies [11,23,37,40]. MMN variables were also not found to be different with respect to gender in the present study which supports the reports from previous similar studies [41,42].

The neural mechanisms contributing to the generation of MMN still remain unclear. Näätänen and Escera (2000) acknowledged two possible theoretical interpretations for MMN [1]. According to the “neural adaptation hypothesis”, repeated presentation of the standards results in adapted (i.e., attenuated) responses of feature-selective neurons in the auditory cortex [43]. Rare deviant sounds activate neurons that are less adapted than those stimulated by the more frequent standard sounds, and thereby elicit a larger response. Another hypothesis, the “sensory memory” or “memory mismatch” account views MMN as a distinct cognitive component of the auditory ERP which arises from the active comparison of current input with a memory trace for recently encountered sounds [44-46].

Studies evaluating MMN variables in infants and children without auditory processing disorders are sparse so far. Most of the studies have investigated a specific group of child population with phonological disorders, dyslexia, autism or other disorders including control groups to compare the results. The present study has evaluated normally developing infants and young children for MMN responses in different sets of experiments in an attempt to optimize the clinical applicability of the MMN values in the age group studied. The features of MMN variables vary with the nature, difficulty and concurrent deviation of multiple stimulus dimensions that have been reported in the present study.

The present research can promote the interpretation of MMN results and the MMN values can be used as a reference not only for those obtained from the classic paradigm but with other discriminable auditory dimensions also. However, as MMN could reflect the whole profile and extent of the central auditory deficits, further scientific research with several auditory attributes and multidimensional deviants in MMN paradigms, in different age groups is still necessitated.

Limitations of the study

The study evaluated the MMN responses for different discriminable auditory dimensions including frequency, intensity and duration. MMN responses to location deviant, however, could not be included in the study. MMN responses for standard-deviant differences (with increment or decrement in deviant stimulus magnitude) for each auditory attribute could have been further recorded and studied in greater detail to obtain a better insight into the features of MMN variables.

## Conclusions

MMN was recordable in all the participants in the present study in all the sets of experiments. The latency of MMN was not found to be different in MMN responses with two-dimensional deviants, nor was the deviant stimulus variation shown to statistically significantly affect the latency. Amplitude changes, however, were statistically significant in different trials. Increased values with an increase in the degree of standard and deviant differences were observed. Also, larger MMN in double deviant responses were evident. The age and gender of the participants were not found to influence the MMN variables in the study.

Standardised MMN variables obtained with MMN paradigms including several auditory attributes in the studied age group can improve the clinical applicability of the test, as particular auditory features may be deranged in different clinical populations. It can be a valuable tool to objectively evaluate the profile of discrimination abilities for different auditory attributes. Further research with different age groups and with additional MMN paradigms to reflect the complete profile of auditory deficits are still warranted.
